# Identification of volatile organic compounds related to the eating quality of cooked *japonica* rice

**DOI:** 10.1038/s41598-022-21863-4

**Published:** 2022-10-28

**Authors:** Yoon Kyung Lee, Su Jang, Hee-Jong Koh

**Affiliations:** Department of Agriculture, Forestry and Bioresources, Plant Genomics and Breeding Institute, Research Institute for Agriculture and Life Sciences, Seoul National University, Seoul, South Korea

**Keywords:** Biochemistry, Plant sciences, Plant breeding

## Abstract

Eating quality (EQ) of rice has a complex nature composed of physicochemical properties. Nevertheless, breeding programs evaluating EQ through sensory test or taste-evaluation instruments have been laborious, time-consuming and inefficient. EQ is affected by both taste and aroma. However, in actual breeding programs, aroma of cooked rice has been considered the least due to lack of information. Here we identified a total of 41 volatile compounds potentially affecting the EQ of non-aromatic, cooked *japonica* rice, identified by GC–MS, sensory panel test, and Toyo taste-meter analyses. Partial least squares discriminant analysis demonstrated an outstanding classification effect of the identified volatile compounds on eating-quality discrimination. Several volatile compounds related to lipid oxidation and fatty acid degradation were identified to affect the EQ in *japonica* rice. Of them, 1-octen-3-ol, 1-ethyl-3,5-dimethylbenzene, 2,6,11-trimethyldodecane, 3-ethyloctane, 2,7,10-trimethyldodecane, methyl salicylate, 2-octanone, and heptanal were selected as important compounds. The discriminant model for the classification of the quality of cultivars was robust and accurate, an r-squared value was 0.91, a q squared value was 0.85, and an accuracy was 1.0. Overall, the results of this study characterize EQ of rice cultivars based on volatile compounds, suggesting the application of metabolite profiling data for rice breeding of high eating quality.

## Introduction

Rice (*Oryza sativa* L.) is one of the most important agricultural crops and serves as a staple food worldwide. Because of improvements in living standards, the market demand for high-quality rice has been increasing^1^. Sensory panel test is a direct method for evaluating the EQ of cooked rice. Preferably gender balanced trained panelists individually rate the cooked rice samples for intensities and preferences of attributes, like appearance, hardness, stickiness, taste or flavor, texture, and overall eating quality^2,3^. Despite of its direct and intuitive evaluation in EQ, sensory panel test is time-consuming, labor-intensive, requiring large volume of samples, so it is not applicable to early generation testing in breeding programs. Therefore, it has been replaced by the analysis of physicochemical properties of rice. Starch is a main component of rice endosperm, which consists of amylose and amylopectin^4^. Hence starch related traits such as amylose content, gel consistency, and gelatinization temperature have been widely studied^5–8^ and their genetic backgrounds have been determined such as granule-bound starch synthase and starch branching enzymes, representatively^9–12^. However, EQ determined by taste-evaluation instruments and physicochemical analysis have not been so satisfying so far. Considering that EQ is a complicated trait, all attributes related to the human sense are significant in discriminating EQ. While the olfactory sense primarily perceives the information, aroma and flavor are considered two of the main EQ-related factors in rice sensory properties^13–15^. Empirically, rice breeders recognize that accurate sensory evaluation of cooked rice is hard to achieve when having a stuffy nose.

Attempts have been made to screen and profile the volatile organic compounds (VOCs) of rice; however, little is known about the relationship between these compounds and the flavor or EQ of cooked rice^16^. Consequently, aroma and flavor have been restrictively considered in application of identified chemical compounds in evaluation of EQ. Moreover, most of the previous studies focused on aromatic rice cultivars, such as basmati and tropical *japonica* rice. For instance, 2-acetyl-1-pyrroline was identified as the VOC responsible for the specific popcorn-like aroma and the characteristic flavor of fragrant rice. In addition, a molecular marker, based on an 8 bp deletion in the fragrance gene, was developed to distinguish between fragrant and non-fragrant rice cultivars^17–19^.

Over 300 volatile compounds have been identified in rice via analytical chemistry. Among these compounds, a few have been identified as oxidation products and are considered as possible negative contributors to rice flavor^20–22^. A recent study aimed to detect volatile compounds in cooked *japonica* rice using solid phase microextraction (SPME) with gas chromatography–resonance-enhanced multiphoton ionization time-of-flight mass spectrometry (GC/REMPI-TOFMS) focused on decreasing the extraction time and comparing the method in detection of particular compounds, 4-vinylphenol and indole^23^. Zhang et al.^24^ performed metabolite profiling via headspace (HS)-SPME GC/MS and HS GC/ion mobility spectrometry (IMS) to discriminate between white and yellow rice using partial least squares discriminant analysis (PLS-DA). Consequently, hexanal, nonanal, octanal, 1-pentanol, and 2-pentyl-furan, involved in fatty acid oxygenation, were identified as compounds with high variable importance in projection (VIP) scores. Thus, further research on VOCs of cooked *japonica* rice cultivar is needed in order to characterize important compounds that affect the general sensory properties, and finally to utilize it for breeding desirable high EQ rice cultivars.

This study aims to identify volatile compounds in cooked rice which significantly affect the EQ of non-aromatic *japonica* rice cultivars in order to provide a fundamental information to set the evaluation standard of EQ for genetic improvement.

## Results and discussion

### EQ evaluation

**Figure 1:**
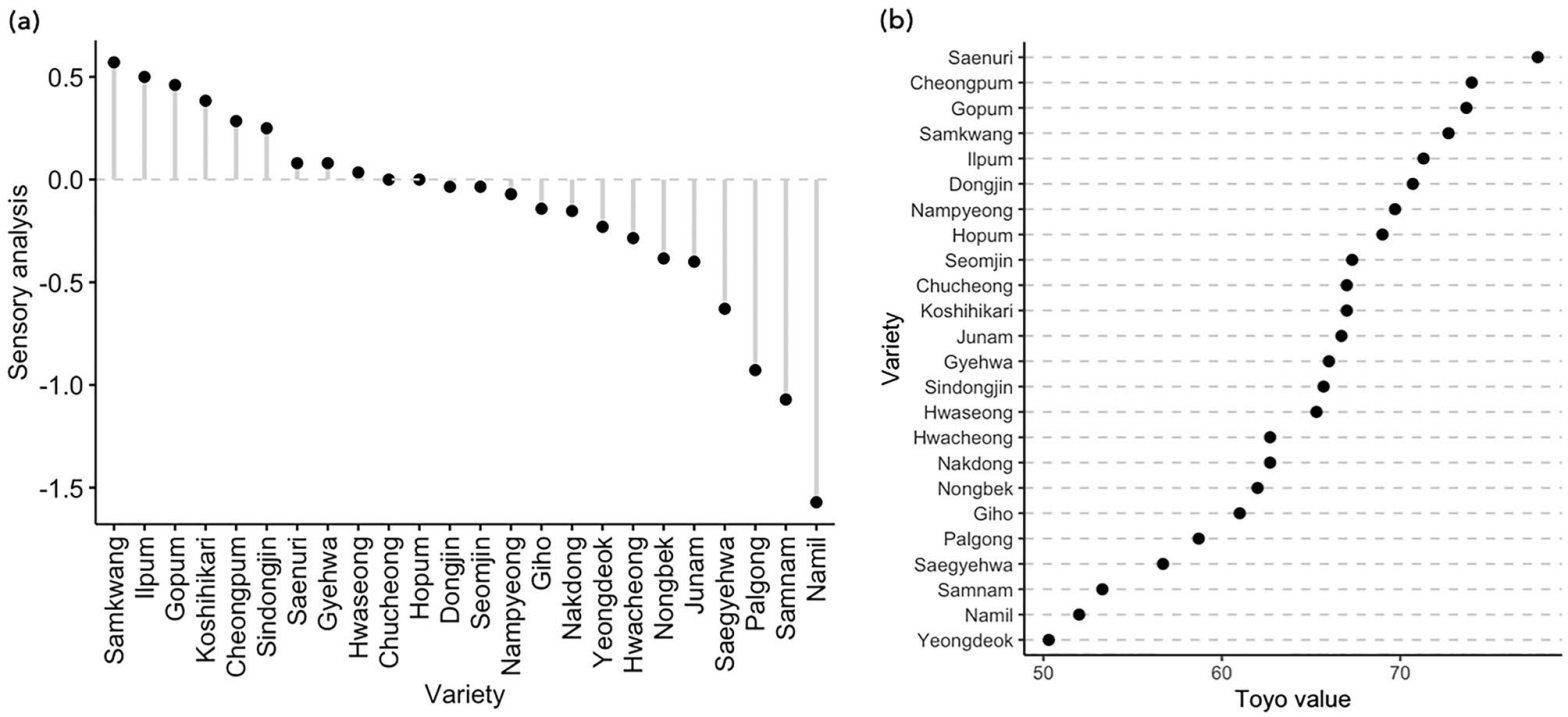
Evaluation of the EQ of 24 rice cultivars using the sensory panel test and Toyo taste-meter. (**a**, **b**) EQ of rice cultivars measured by the sensory panel test (**a**) and Toyo taste-meter (**b**).

The EQ of 24 non-aromatic temperate *japonica* rice cultivars was evaluated in this study, based on the sensory panel test and Toyo taste-meter readings (Fig. 1). According to the results of the sensory panel test conducted by 14 trained panelists, Samkwang was the best EQ cultivar, followed by Ilpum, Gopum, Koshihikari, and Cheongpum. The least favored cultivars were Namil, followed by Samnam and Palgong. Meanwhile, EQ measured by Toyo taste-meter was the highest in Saenuri followed by Cheongpum, Gopum, Samkwang, and Ilpum. On the other hand, Yeongdeok showed the lowest Toyo taste-value, followed by Namil, Samnam, Saegyehwa, and Palgong. A study conducted by Lestari et al*.*^25^ evaluating the eating quality of 22 *japonica* rice used Toyo taste-values in marker-trait regression analysis. The used cultivars were not completely identical, but Gopum, Samkwang, Ilpum, and Koshihikari exhibited high Toyo taste-value above 70. Similarly, low Toyo taste-value was observed around 60 in Palgong and Samnam in the study. Another kind of taste-evaluation instrument, Satake taste analyzer (Satake, STA1B-RHS1A-RFDM1A, Japan) generates taste score of cooked rice for EQ evaluation^26–28^, and was successively used in evaluating 533 rice accessions for the genome-wide association study^29^. Although there was a strong correlation between EQs by the sensory panel test and Toyo taste-meter (see Supplementary Fig. S1 online), Toyo taste-meter which is widely used to evaluate the EQ of cooked rice for its relative convenience in comparison with sensory test^30,31^ may not accurately evaluate the EQ of cooked *japonica* rice. The differences in ranking by two methods could be explained by the fact that Toyo taste-meter only consider the glossiness of the surface of cooked rice grains. This suggests that flavor and aroma should be considered together to overcome the limit of the conventional EQ evaluation methods like Toyo taste-meter which measures and generates the numerical value by the appearance of cooked rice.

### Identification of volatile compounds in cooked rice

Based on the results in Fig. 1, seven cultivars with higher EQ (Samkwang, Ilpum, Gopum, Koshihikari, Cheongpum, Sindongjin and Saenuri), and seven cultivars with lower EQ (Namil, Samnam, Palgong, Nongbek, Hwacheong, Yungdeok, and Giho) compared to the check variety, were selected for analysis of the volatile compounds that affect the EQ of rice. The cooked rice of 14 *japonica* cultivars was analyzed using HS-SPME GC–MS/MS, and 41 volatile compounds were identified as Table 1 and quantified (see Supplementary Table S1 online). These compounds were classified into nine different classes: alcohols, aldehydes, carboxylic acid, ester, hydrocarbons, imine, ketones, phenols, terpenoid, and unknown compounds. The most frequently observed classes were hydrocarbons and aldehydes. Among the hydrocarbons, alkanes and alkenes are reportedly derived from lipid breakdown. Nonadecane was previously detected in aromatic and non-aromatic rice cultivars^32^. Although a number of alkanes and alkenes were detected in the current study, limited information is available on their effects on the flavor of cooked rice. Aldehydes usually exhibit a relatively low odor threshold and are considered one of the main factors affecting the overall flavor profile of cooked rice. Among the identified aldehydes, hexanal exhibits fruity, grass, and green attributes; however, lipid oxidation generates a large amount of hexanal, resulting in off-odors in rice^33–35^. Octanal, heptanal, and nonanal are derived from oleate hydroperoxide decomposition. Moreover, low odor threshold alcohols, such as lipid-derived 1-octen-3-ol and polyunsaturated fatty acid metabolism-related 1-hexanol, were also identified. All identified volatile compounds were subjected to further statistical analysis to identify important volatile features related to the EQ of cooked temperate *japonica* rice.

**Table 1:** Volatile compounds identified by HS-SPME GC/MS.

ID	Compound name	Retention time (min)	Retention index
1	Decane	13.88	Below 1000
2	2,7,10-Trimethyl-dodecane	15.23	1027
3	3-Ethyloctane	16.38	1052
4	2,6,11-Trimethyl-dodecane	17.63	1079
5	Hexanal	18.51	1098
6	Dodecane	23.42	1200
7	Heptanal	23.56	1203
8	Unknown_71	25.03	1234
9	Unknown_57	27.77	1291
10	Nonadecane	28.21	1300
11	2-Octanone	28.37	1304
12	Octanal	28.60	1309
13	Unknown_69	29.83	1335
14	(E)-2-Heptenal	30.48	1349
15	6-Methyl-5-hepten-2-one	30.89	1358
16	1-Hexanol	31.14	1363
17	2-Methyl-1-pentadecene	31.14	1364
18	1,3,5-Trimethyl-benzene	31.29	1367
19	1-Ethyl-3,5-dimethyl-benzene	31.89	1380
20	1-Methyl-4-(1-methylethyl)-benzene	32.23	1387
21	2-Ethyl-1,4-dimethyl-benzene	32.60	1395
22	Nonanal	33.47	1414
23	Unknown_57(2)	34.09	1428
24	Unknown_43	34.73	1443
25	1,3-Bis(1,1-dimethylethyl)-benzene	34.87	1446
26	1,2,3,5-Tetramethyl-benzene	35.32	1456
27	1-Octen-3-ol	35.51	1461
28	3-Methyl-tetradecane	35.60	1463
29	1,2,3,4-Tetramethyl-benzene	35.82	1467
30	2-(3-Methylbutyl)-thiophene	36.65	1486
31	1-Octanol	40.24	1566
32	2,6,10,15-Tetramethyl-heptadecane	41.77	1600
33	4-Methyl-benzaldehyde	46.74	1685
34	Methoxy phenyl oxime	51.57	1760
35	Methyl salicylate	54.96	1814
36	trans-Calamenene	57.74	1865
37	6,10-Dimethyl-5,9-undecadien-2-one	58.37	1876
38	2-methyl-propanoic acid, 1-(1,1-dimethylethyl)-2-methyl-1,3-propanediyl ester	59.25	1893
39	Butylated hydroxytoluene	61.27	1935
40	2-Methoxy-5-vinylphenol	74.41	2232
41	2,4-Bis(1,1-dimethylethyl)-phenol	77.72	Over 2300

### Statistical analysis of the EQ-related compounds

**Figure 2:**
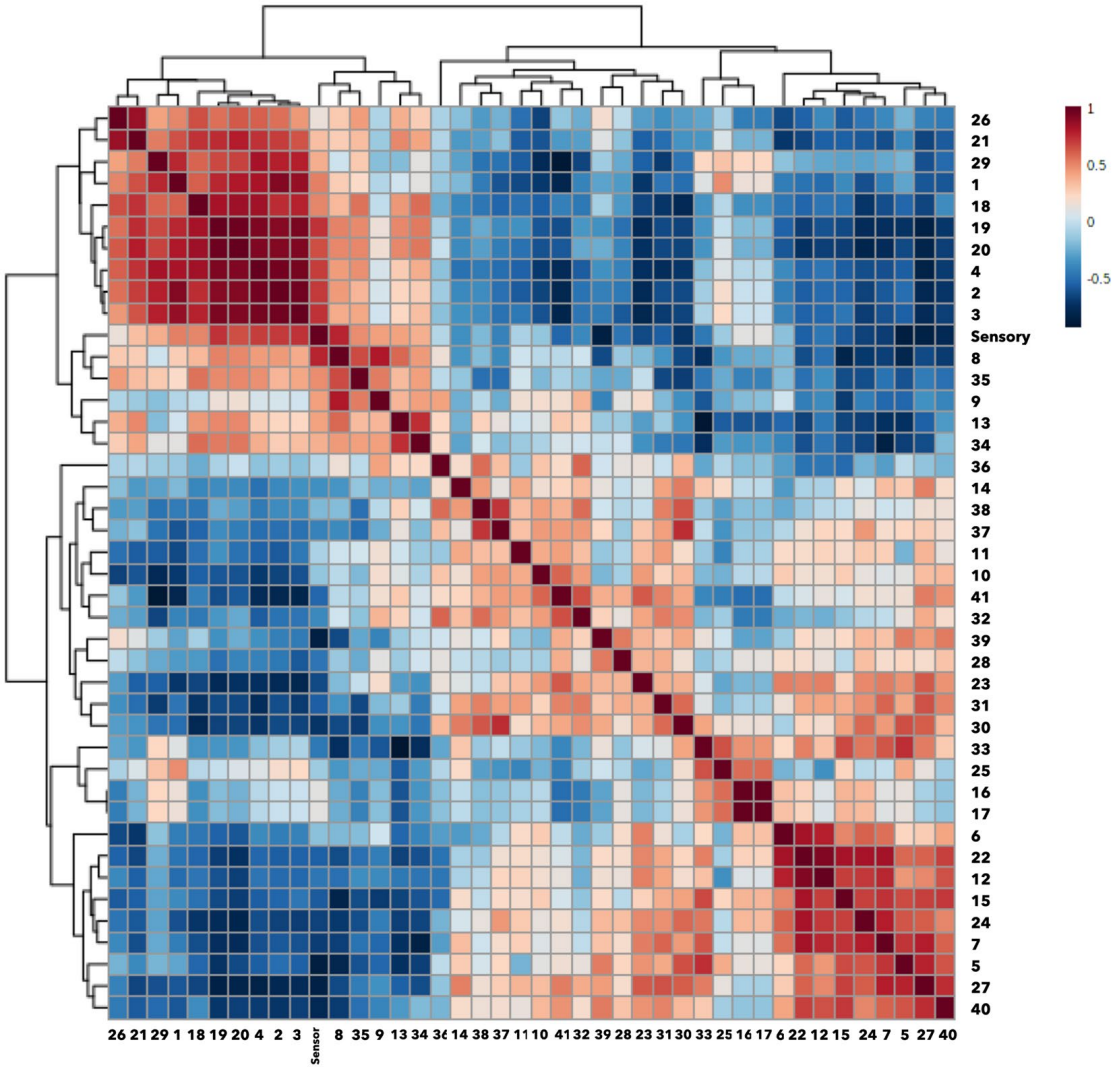
Pearson’s correlation analysis of the sensory panel test scores and HS-SPME GC/MS-identified volatile compounds. The ID numbers of VOCs are indicated. The color scale indicates the correlation coefficients ranging from 1 (red) to -1 (blue).

Prior to data analysis, the relative peak area ratio and sensory panel test score of each compound were normalized and scaled as shown in Supplementary Figs. S1 and Fig. S2 online. Pearson’s correlation analysis was performed to identify volatile compounds highly correlated to the sensory panel test result (Fig. 2).

Of the 41 compounds, seven compounds, 3-ethyl-octane, 1-ethyl-3,5-dimethylbenzene, 2,7,10-trimethyldodecane, 2,6,11-trimethyldodecane, 1-methyl-4-(1-methylethyl)-benzene, decane, and 1,3,5-trimethylbenzene, showed significant positive correlations. Numerous benzene-derived aromatic hydrocarbons were previously identified in unprocessed rice samples^36^. Decane was previously identified in cooked scented-rice samples^37,38^. The above-mentioned volatile compounds have been identified in cooked non-aromatic rice for the first time. Ten of the 41 compounds, hexanal, butyl hydroxytoluene, 1-octen-3-ol, 2-methoxy-5-vinylphenol, 2-(3-methylbutyl)-thiophene, heptanal, 6-methyl-5-hepten-2-one, 1-octanol, nonanal, and 2,4-bis(1,1-dimethylethyl)-phenol, showed significant negative correlations with the sensory panel test. Aliphatic aldehydes, such as hexanal, heptanal, and nonanal, are generated from the degradation of fatty acids, and 1-octen-3-ol is a well-known lipid-derived alcohol^34,39,40^. Thus, these lipid oxidation products could have negatively affected the EQ of rice.

**Figure 3:**
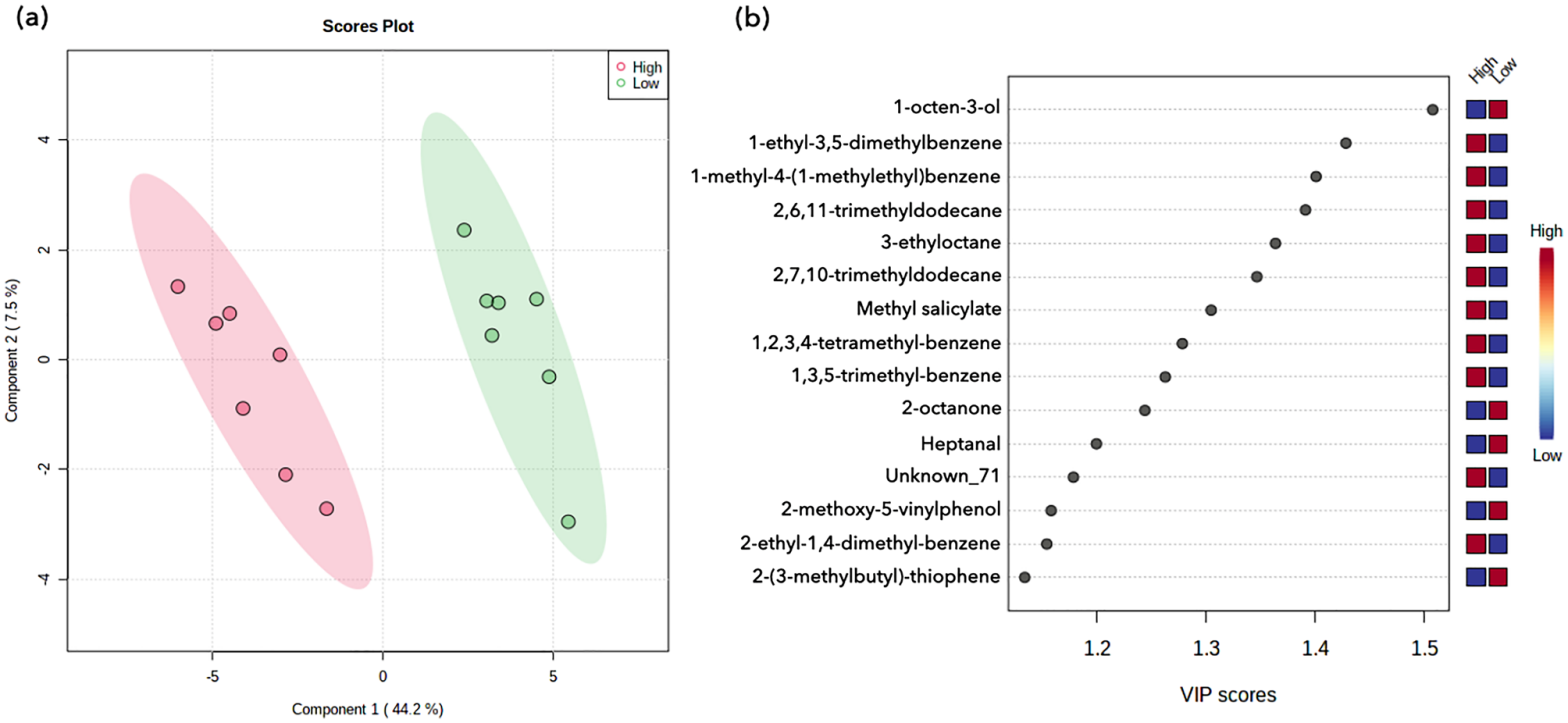
PLS-DA of the HS-SPME GC/MS data of non-aromatic rice cultivars. (**a**) PLS-DA score plot. (**b**) VIP scores of compounds. The color-coded boxes indicate peak area ratio as high (red) and low (blue), first column is high eating-quality cultivars, and second column is low eating-quality cultivars.

The PLS-DA models were used to discriminate between high and low EQ cultivars, based on the volatile compounds identified in cooked rice samples. The first latent variable explained 44.2% of the total variables (Fig. 3a). The score plot indicated a clear segregation of rice cultivars based on their EQs. This implies that the detection method and the identified volatile compounds were appropriate for the identification of rice cultivars with superior EQ. The accuracy, goodness of fit, and goodness of prediction of this model were 1.0, 0.911, and 0.846, respectively, when the number of components was 1. These values signify that the model generated in this study is both accurate and robust.

The variable importance in projection (VIP) scores, which imply biomarkers that play important roles in the discrimination from the PLS-DA model, were also calculated (Fig. 3b). Among the volatile compounds, 1-octen-3-ol displayed the highest VIP score (1.51), followed by 1-ethyl-3,5-dimethylbenzene, 1-methyl-4-(1-methylethyl)benzene, 2,6,11-trimethyldodecane, 3-ethyloctane, 2,7,10-trimethyldodecane, methyl salicylate, 2-octanone, and heptanal. Notably, the VIP scores of lipid oxidation products were higher than 1. Additionally, comparatively higher peak ratio of 1-octen-3-ol was detected through out of low EQ cultivars (see Supplementary Fig. S2 online), which implies that 1-octen-3-ol potentially negatively affected on the EQ. On the other hand, higher peak ratios were shown from the benzene-derived aromatic hydrocarbons, 3-ethyloctane, and methyl salicylate in high EQ cultivars. These compounds can be considered as the VOCs that are positively related to the EQ. The higher the VIP scores, the clearer segregation patterns of the peak ratios between the low and high EQ cultivars were observed. The results imply that the content of certain VOCs could provide supplementary information regarding flavor and aroma in evaluation of EQ. A recent study on the evaluation of EQ of 6 japonica rice varieties presented that JR5, one of the varieties considered as high-EQ in the study, had higher 1-octen-3-ol and explained it as positively contributed VOC to the aroma of the variety^41^. However, results of the study lack statistical power and failed to present a scientific basis on the effects of the VOCs on determining EQ. On the other hand, the PLS-DA model of this study clearly presented and suggested VOCs that positively or negatively affect EQ.

Meanwhile, there still is grey area to practically utilize the results, for instance the interactions among the VOCs should be further studied and considered. Furthermore, an unknown compound that potentially affects the EQ of rice should be investigated further and identified. Either positively or negatively affect, the compounds with VIP scores greater than 1 are considered as key VOCs affecting the general sensory properties of cooked *japonica* rice. These could further be used as quality evaluation criteria to consider aroma and flavor attributes in rice breeding programs.

## Conclusion

In this study, the EQ of 14 non-aromatic *japonica* rice cultivars was evaluated using the sensory panel test, Toyo taste-meter, and volatile compound profiling. A number of volatile compounds were identified for the first time in cooked non-aromatic *japonica* rice, and these compounds showed a strong correlation with the EQ of rice. Notably, lipid-derived compounds (e.g., 1-octen-3-ol), fatty acid degradation-related compound (heptanal), and other compounds (e.g., 2-octanone, methyl salicylate, and other benzene-derived compounds) were identified as important variables that discriminate rice cultivars based on EQ. To take into account the aroma and flavor characteristics of cooked rice in evaluating its EQ, a highly accurate discriminant model was generated. The contents of listed significant VOCs could be suggested for the new EQ standard. The results could serve as a foundation for future research on integrated rice EQ, and could facilitate the development of high-quality rice varieties.

## Materials and methods

### Plant material and growth conditions

A total of 24 non-aromatic temperate *japonica* rice cultivars were selected, based on prior knowledge of their putative EQ: Koshihikari, Chucheong, Gopum, Samkwang, Sindongjin, Ilpum, Saenuri, Cheongpum, Gyehwa, Dongjin, Seomjin, Hwaseong, Nampyeong, Hopum, Yeongdeok, Giho, Nakdong, Nongbek, Hwacheong, Samnam, Palgong, Junam, Saegyehwa, and Namil. All the accessions were retained at the Agricultural Genetic Resource Center, Seoul National University, Suwon, South Korea, and followed the relevant institutional guidelines.

All rice cultivars were cultivated in 2020 in an experimental farm of Seoul National University located in Suwon. The general lowland cultivation method was applied. The heading date of each cultivar was recorded, and plants were harvested in 45–50 days after heading. The harvested plants were air dried until reaching the grain water content of 13–14%, and subsequently threshed using a thresher. The grains were dehulled (using a dehull machine), milled to 92.2% (using a milling machine), and immediately stored in the low-temperature storage at 12 °C until the experiment.

### Sensory panel test

Samples were prepared and cooked according to the protocol of the National Institute of Crop Science (NICS), Rural Development Administration (RDA), Korea^42^. The milled rice samples were weighed to 200 g, washed five times with tap water, and soaked for 20 min. The water was drained off for 10 min, and the macerated rice was cooked in 1.2 volumes of water (rice: water = 1:1.2 w/w) using the automatic cooking cycle of an electric rice cooker. Subsequently, the cooked rice was mixed thoroughly in the rice cooker and allowed to sit for 15 min. Sensory evaluation was performed by a panel of 14 trained members. The EQ of each cultivar was scored from + 2 (very good) to − 2 (very bad) in comparison with that of Chucheong (reference sample; score = 0), and the average value was computed for each cultivar.

### Toyo taste-meter analysis

The Toyo taste-meter (MA-30A; Toyo Rice Corporation, Wakayama, Japan) was used to measure the EQ of cooked rice. The Toyo taste-value is known to be significantly correlated with the palatability of cooked rice^43^. Head rice (33 g) was cooked at 80 ℃ for 10 min, and then allowed to sit at room temperature for 5 min. The glossiness of the surface of the cooked rice grains was measured in triplicate using certain electromagnetic waves, and then converted into the Toyo taste-value.

### Untargeted profiling of volatile compounds

The milled rice samples were cooked according to the NICS protocol, with some modification. Briefly, milled rice samples (3 g) were quantified, washed, and soaked in tap water for 20 min. After draining the water, each rice sample was transferred to a 20 ml glass vial (CTC, Perkin Elmer & Agilent), to which 3.6 ml distilled water and 2 μl of 2000 ppm 1,2,3-trichloropropane (Sigma-Aldrich, St. Louis, MO; internal standard) were added. The vials were closed tightly with a magnetic crimp cap using PTFE/silicone septa, and vortexed vigorously. Then, the sealed glass vials were placed in an electronic rice cooker, and samples were cooked for 25 min.

VOCs were analyzed using the HS-SPME injector-equipped Thermo Scientific Trace 1310 Gas Chromatograph, TSQ 8000 Triple quadrupole Mass Spectrometer and TriPlus RSH autosampler (Waltham, MA, USA) with a DB-Wax capillary column (60 m × 0.25 mm, 0.50 μm film thickness; Agilent Technologies). The samples were incubated for 10 min at 70 °C. Headspace volatiles of the cooked rice sample were adsorbed by inserting DVB/CAR/PDMS StableFlex SPME fiber (2 cm, 50/30 μm; Supelco, Bellefonte, PA, USA) into the vial for 50 min at 70 °C with agitation, and then desorbed for 2 min. Blanks were run after every 10 samples as a control. The injector temperature was 250 °C. The oven temperature of GC was as follows: initial temperature of 40 °C held for 2 min; increased to 150 °C at 3.0 °C/min and held for 10 min; increased to 200 °C at 3.0 °C/min and held for 5 min; and increased to 230 °C at 6.0 °C/min and held for 5 min. Research-grade helium was used as the carrier gas at a constant flow rate of 2.0 ml/min in the splitless with purge mode. The mass spectrometer was set to the electron impact mode at 230 °C with 70 eV and scanned at 35–550 m/z. Analysis was performed in triplicate. The compounds were identified by comparing the chromatogram and retention indices with the reference in the database, NIST Mass Spectral Search Program for the NIST/EPA/NIH Mass Spectral Library, version 2.0g (National Institute of Standards and Technology, Gaithersburg, MD, USA), with a match score cutoff of at least 80%. The data obtained were processed using the Xcalibur software (Thermo Fisher Scientific, Waltham, MA, USA). The ion peak area of the identified compound was divided by that of 1,2,3-trichloropropane (internal standard), and the calculated area ratio was used for further statistical analysis (see Supplementary Table S1 online).

### Statistical analysis

All data were presented as mean values. Pearson’s correlation and Spearman correlation analyses were performed using a package from the RStudio 1.1.453 software (R Foundation for Statistical Computing, Vienna, Austria). To perform multivariate statistical analysis, the relative peak area of the identified volatile compounds was normalized to quantile, scaled to mean centered, and divided by the standard deviation of each variable (see Supplementary Figs. S3 and S4 online). The resultant data were then subjected to PLS-DA using MetaboAnalyst 5.0^44^.

## Supplementary Information

Supplementary Figures.

Supplementary Table S1.

## Data Availability

Datasets generated in the current study are available from the corresponding author upon reasonable request.

## Abbreviations

HS-SPME: Headspace solid phase microextraction
GC/MS: Gas chromatography mass spectrometry
VOCs: Volatile organic compounds
EQ: Eating quality
PLS-DA: Partial least squares discriminant analysis
VIP: Variable importance in projection
NICS: National Institute of Crop Science
RDA: Rural Development Administration
