# Supplementary material for: Identification of volatile organic compounds related to the eating quality of cooked japonica rice
Source: Sci Rep. 2022 Oct 28;12:18133. doi: 10.1038/s41598-022-21863-4 (PMC9616908; doi:10.1038/s41598-022-21863-4)
Supplement: Supplementary file 1 — Supplementary Figures. [file 41598_2022_21863_MOESM1_ESM.docx]

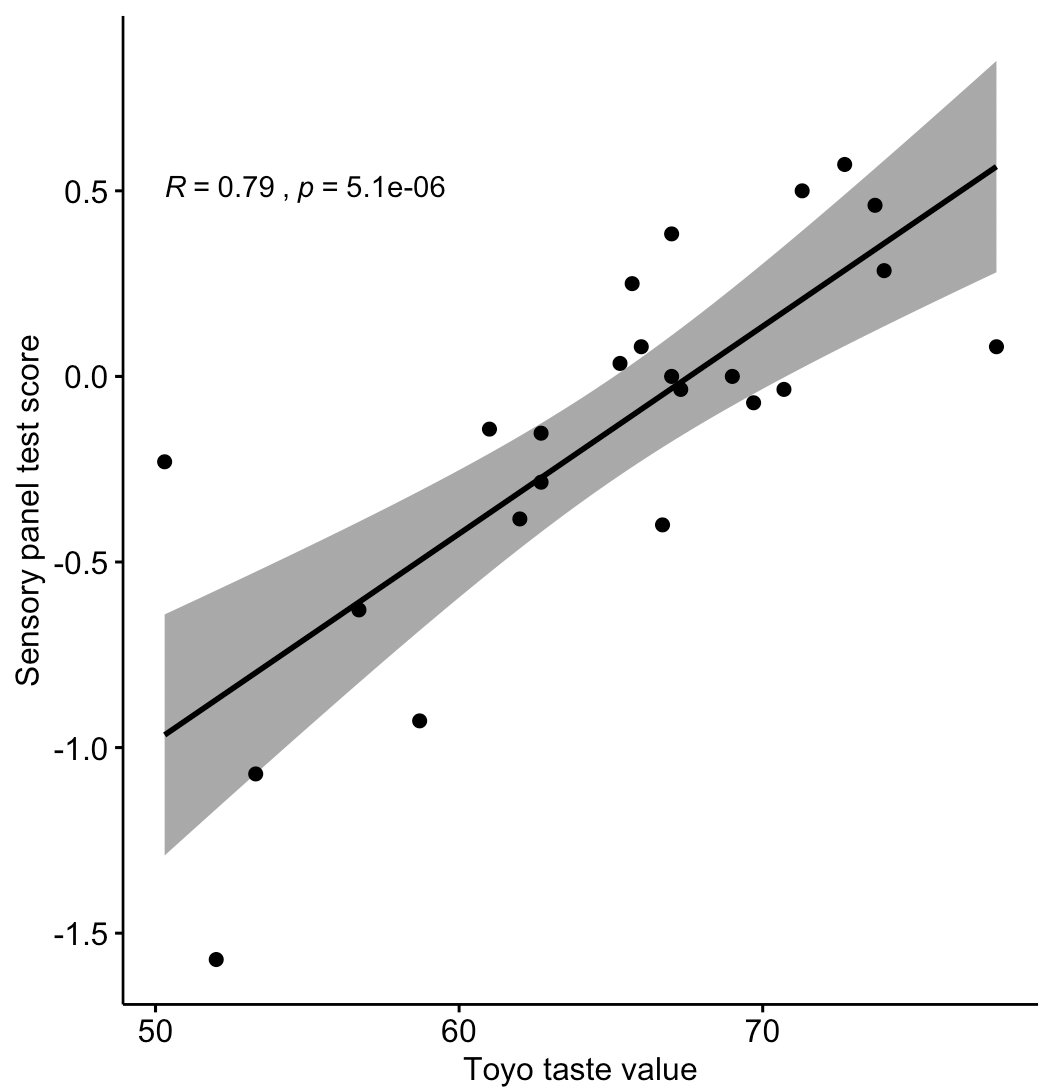


Supplementary Figure S1. Rank correlation analysis of Toyo taste-meter readings and sensory panel test results.


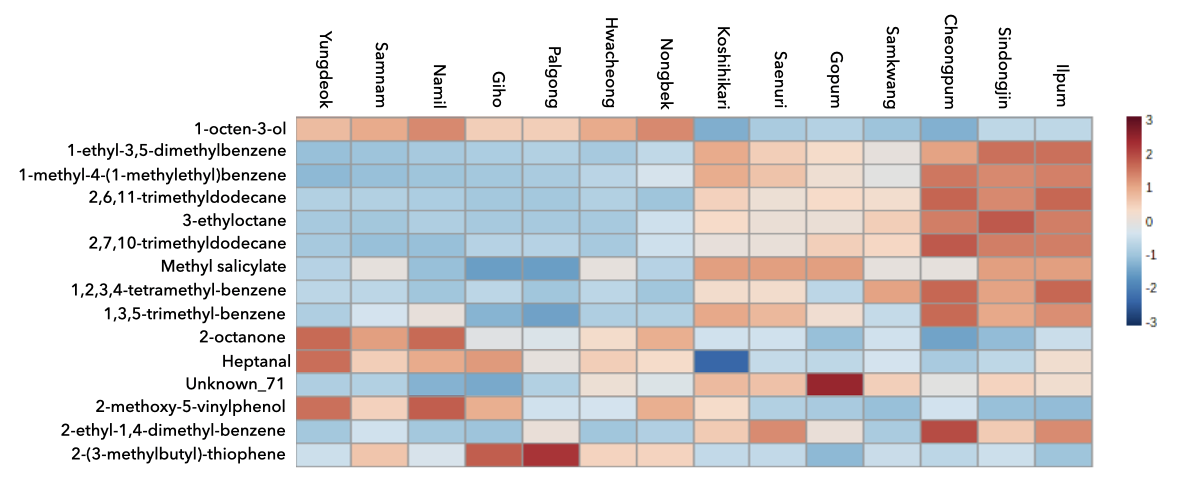


Supplementary Figure S2. Heatmap analysis of the relative peak ratio of important VOCs according to the cultivars. The color code indicates the differences in the relative peak ratio of significant VOCs, ranging from blue (negative) to the red (positive).


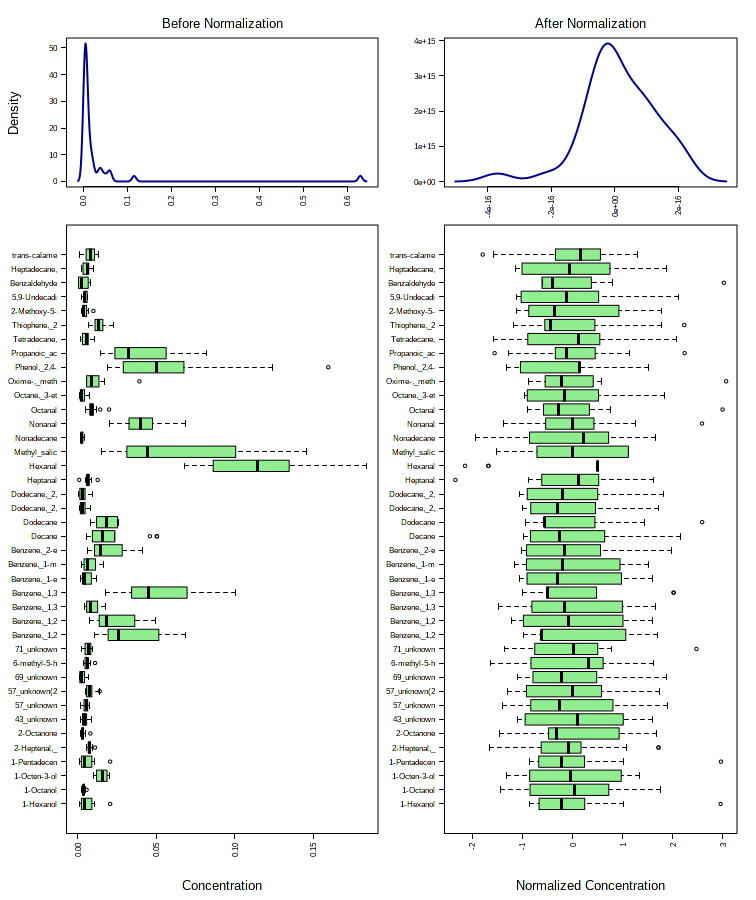


Supplementary Figure S3. Normalized and scaled peak area ratio of identified VOCs


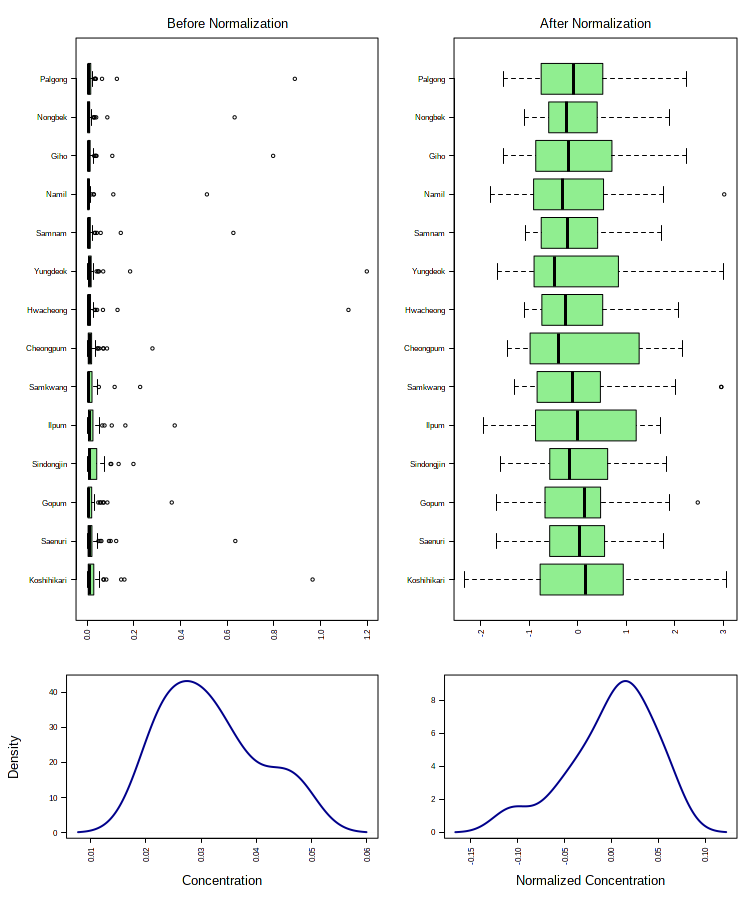


Supplementary Figure S4. Normalized and scaled sensory panel test scores.
